# The Correlation Between Non-Invasive Ventilation Use and the Development of Dry Eye Disease

**DOI:** 10.7759/cureus.18280

**Published:** 2021-09-25

**Authors:** Priya V Shah, Lawrence Zhu, Anjum Kazi, Angela Zhu, Aleksander Shalshin

**Affiliations:** 1 Biomedical Sciences, New York Institute of Technology College of Osteopathic Medicine, Old Westbury, USA; 2 Natural Sciences and Mathematics, State University of New York (SUNY) Cobleskill, Cobleskill, USA; 3 Pulmonary Medicine, Plainview Hospital, Plainview, USA

**Keywords:** obstructive sleep apnoea, cpap, non-invasive ventilation, dysfunctional tear syndrome, keratoconjunctivitis sicca, dry eye disease, dry eyes

## Abstract

The use of non-invasive ventilation (NIV) devices such as continuous positive airway pressure and bi-level positive airway pressure machines have been associated with an increased incidence of dry eye disease (DED). To understand how the use of these ventilation masks impacts the eyes, a review of the pathophysiology of DED and an evaluation of recent studies investigating the effects of NIV use on the severity and incidence of this condition were performed. It was found that the use of face masks associated with the ventilation devices exhibited a positive correlation to the incidence and severity of numerous ocular pathologies. However, the benefits of non-invasive mechanical ventilation are undeniable in treating conditions such as obstructive sleep apnea, chronic obstructive pulmonary disease, and respiratory failure; therefore, proper education, behavioral modifications, and treatment can help reduce or prevent the adverse effects that NIV have on the eyes.

## Introduction and background

Non-invasive ventilation (NIV) was initially used to treat patients with acute respiratory failure in the early 1940s; however, in the last two to three decades, substantial evidence on the efficacy of NIV in treating severe obstructive sleep apnea (OSA) and exacerbations of chronic obstructive pulmonary disease (COPD), congestive heart failure (CHF) induced pulmonary edema, and acute and chronic hypoxic and hypercapnic respiratory failure has been documented [1]. In 1980, Dr. Colin Sullivan, an Australian physician, invented the continuous positive airway pressure (CPAP) NIV device in treating sudden infant death syndrome (SIDS) and upper respiratory upper airway pathologies (e.g., OSA). OSA is a disorder caused by a decrease or complete cessation of airflow when one is sleeping. During sleep, muscle relaxation leads to soft tissue in the back of the throat to collapse and obstruct the upper airway. Prior to the invention of the CPAP device, severe OSA was treated with an invasive procedure known as a tracheotomy (a surgical opening created through the neck into the trachea). With the use of NIV such as CPAP, the obstruction is minimized and the airway remains patent during sleep by providing an increase in air pressure in the trachea. CPAP remains the first-line treatment for severe OSA [2].

However, there are side effects associated with the long-term use of these devices, which have largely contributed to the low patient compliance in CPAP therapy. The overall rate of CPAP non-adherence based on seven hours of sleep a night has been reported to be as high as 34.1% [2]. While the most apparent symptoms associated with CPAP usage are exhalation discomfort, aerophagia (air swallowing), dry and stuffy nose, and dry mouth, people often fail to mention dry eyes. The acceleration of ocular drying with the use of NIV devices can worsen or cause dry eye disease (DED). DED, also known as keratoconjunctivitis sicca or dysfunctional tear syndrome, is the most common ocular surface disorder worldwide. It has a varying prevalence of 5% to 50% [3]. DED typically presents with symptoms including, but not limited to, foreign body sensation, grittiness, itchiness, and visual disturbances. There are a plethora of risk factors that can contribute to the disease, such as normal aging, inflammatory conditions, systemic medications, environmental factors, and lifestyle, but studies show that there has been a significant increase in cases of eye irritation with the use of NIV devices [4]. We sought to review the pathophysiology of DED and better understand the association between NIV and the development of DED. Articles examining the incidence and prevalence of DED and dry eye symptoms in patients treated with NIV and behavioral modifications that can help reduce this ophthalmic adverse effect were reviewed.

## Review

Pathophysiology of keratoconjunctivitis sicca (dry eye disease)

DED is a multifactorial disorder of the tear film and ocular surface that can be divided into two categories: aqueous deficient DED and hyper-evaporative DED. Aqueous deficient DED is due to decreased tear production, and evaporative DED is due to abnormal meibomian gland physiology. Although one may predominate, the combination of the two along with other external factors disturbs the microenvironment and disrupts homeostasis of the ocular surface [3].

The lacrimal functional unit includes the ocular surface, meibomian glands, the main lacrimal gland, and the nerves that innervate them [5]. Continuous tear secretion allows for the ocular surface to be lubricated, maintaining comfort and overall conjunctival and corneal function. Tear film is produced by the conjunction of the lacrimal gland, meibomian glands, goblet cells, and many accessory glands that release integral components of the tear film. Each unit ensures the stability of the tear film, and disruption of any unit often results in dry eyes [6]. Stress to the ocular surface, such as dry drafty environments, triggers proinflammatory chemokines, matrix metalloproteinases, and cytokines to activate CD4 T cells. The CD4 cells invade the ocular surface and the lacrimal gland, leading to apoptosis. This creates a vicious cycle of ocular surface damage and inflammation [5].

Damage to the ocular surface can cause significant distress as it is extremely uncomfortable, affecting one’s quality of life, and may hinder daily activities [7]. DED has become increasingly more prevalent in recent years for a plethora of reasons such as wearing contact lenses, rising usage of smartphones and computers, and normal aging [3]. Additionally, environmental factors including cigarette smoke, dust, and exposure to dry air have been shown to worsen DED [8]. In particular, studies have revealed that patients with OSA undergoing CPAP therapy present with a greater risk of eye irritation due to leakage of air from their CPAP mask, creating a dry drafty environment [9]. As the etiology of DED can be attributed to many different factors and may present in various ways, it is imperative to monitor OSA patients for ocular manifestations. Alteration of ocular surface anatomy in patients with OSA undergoing positive airway pressure therapy can lead to serious damage to the surface.

Literature review

Globally, the prevalence of DED has been measured to be 5-50%, with as many as 16 million people affected in the United States alone [3,10,11]. The likelihood of DED increases with the use of NIV devices [12], such as powered air-purifying respirators, CPAPs, and bilevel positive airway pressure (BiPAPs), by blowing air around the face and eyes. To evaluate the effects of NIV on the development of DED, national databases such as PubMed and Google Scholar were searched using the term "non-invasive ventilation and dry eyes" to identify original studies that reported subjective and objective ophthalmologic outcomes, specifically DED, associated with NIV therapy. Review articles were excluded from this review. Publication dates were not limited to provide an adequate review of the literature available. Ancestry searches using the references from selected articles were also performed. A summary of the studies reviewed is presented in Table 1.

**Table 1:** Studies investigating the effects of NIV therapy on the development of dry eye disease. CPAP, continuous positive airway pressure; N-CPAP, nasal continuous positive airway pressure; DED, dry eye disease; EK, exposure keratopathy; IR, incidence rate; n, number of patients; NIV, non-invasive ventilation; NMT, nasal mask therapy; OSA, obstructive sleep apnea; PR, prevalence rate; TBUT, tear breakup time

Author, year	Population	Ventilation type	Condition treated	Outcome findings
Hayirci et al., 2012 [13]	n = 40; 45-61 years old	CPAP	OSA	Increase in Nelson impression cytology grade and decrease in TBUT are in favor of preocular tear film abnormality. Increase in Schirmer 1 score supports the opinion of increased ocular surface irritation.
Kousha et al., 2018 [14]	Total n = 371; first phase, n = 257 (without eye care protocol); Second phase, n = 114 (with eye care protocol); 45-79 years old	Mechanical ventilation	Pulmonary disease	The rate of EK, secondary to corneal dryness, in mechanically ventilated patients was 21% in the first phase of the study. Implementation of the eye care protocol in the second phase of the study significantly reduced occurrence rates to 2.6% (three cases).
Matossian et al., 2020 [12]	n = 350,420; 41.3-64.9 years old	CPAP	OSA	PR and IR of DED were higher than the reported prevalence of DED in the general population. Patients with at least 36 months of CPAP or NMT use had much greater PR of DED (13.0% vs 6.7%) than adults in the general U.S. population and greater IR of DED (10.3% vs 0.6% - 0.9%) than adults in the general U.S. population.
Pépin et al., 1995 [15]	n = 193; 47-71 years old	N-CPAP	OSA	It was found that 50% of patients complained of at least one adverse effect including ocular dryness, gingival discomfort, facial allergy, air leakage, and nasal ridge abrasion. Air leaks near the eyes and over the face were frequent and often led to red eyes in the mornings. This was seen less in patients using individually molded masks (9% vs 24% for others; p < 0.025).
Smith et al., 2019 [16]	n = 50; 59-85 years old	NIV	Hypercapnic respiratory failure	It was reported that 44% of patients experienced dry eyes.
Yesilbalkan and Ozbudak, 2019 [17]	n = 40; 65.5-81.3 years old	NIV	Pulmonary disease	Throughout the seven-day follow-up period, the rate of eye dryness in the patients ranged between 65% and 70%.

While CPAP remains the treatment of choice for patients with OSA [18], many patients are non-compliant due to its side effects. Pépin et al. [15] studied the adverse reactions of CPAP machines in 193 patients in two different sleep centers in France (Lyon and Grenoble). All patients underwent polysomnography to ensure that CPAP pressure settings were adjusted to levels required to sufficiently abolish apneas and restore sleep quality. Subjective effects of CPAP use and objective compliance with treatment were assessed. Pépin found that 50% of patients complained of at least one adverse effect of CPAP usage, regardless of mask type (tightly fitted, individually molded, or silicone), including ocular dryness, gingival discomfort, facial allergy, air leakage, and nasal ridge abrasion. Ocular dryness in CPAP users is due to air leaks over the face which frequently causes eye redness upon awakening. Of patients with silicone masks, 24% experienced ocular dryness compared to the 9% with individually molded masks (p < 0.025). The significant decrease in dryness experienced by patients with individually fitted masks is most likely due to the decreased airflow up toward the eyes throughout the night. The study found that not only eye dryness, but all side effects associated with CPAP usage exhibited a decrease in frequency with the use of individually fitted masks compared to standard masks that allow the escape of air.

Hayirci et al. [13] evaluated the effect of CPAP treatment in patients with OSA on the ocular surface. In a prospective, sectional cohort study, 40 patients with OSA were examined. Patients with a history of previous CPAP therapy, ocular surgery, and other ophthalmologic pathologies including DED were excluded from the study. Each patient was given routine ophthalmologic examinations and ocular surface evaluations, including biomicroscopy, Schirmer 1 testing, tear breakup time measurements, ocular surface staining, and conjunctival impression cytology. These evaluations were performed before and four months after starting CPAP therapy. Hayirci et al. noted an increase in Schirmer 1 scores by an average of 3.20 mm (t = 3.20, p = 0.008). Schirmer’s test is an effective method to measure tear production by placing a piece of filter paper inside the lower lid of the eye and measuring moisture content after five minutes. The increase in Schirmer 1 scores supports the hypothesis that CPAP use induces ocular irritation. In the acute phase, tear production increases due to excessive drying and irritation overnight. Chronically, desiccating stress alters meibum composition, resulting in irregular meibocyte differentiation, and depletes meibocyte stem cells. This depletion leads to hyposecretion and atrophy of the gland, resulting in dry eyes [19].

NIV is also commonly used to manage acute and acute-on-chronic respiratory failure due to COPD and decompensated CHF. In a prospective, cross-sectional study, Smith et al. [16] observed 50 patients admitted to the general medical floor (non-intensive care unit [ICU]) of a tertiary teaching hospital in Australia. These patients were all treated with NIV for hypercapnic respiratory failure. At admission, each patient’s symptomatic burden was measured subjectively using the Condensed Memorial Symptom Assessment Scale (CMSAS) [20]. The CMSAS assessed the severity of 11 physical symptoms present in the last 72 hours. Smith et al. also asked the patients to rate the severity of four additional symptoms typically associated with NIV therapy: cough, sputum production, dry eyes, and gastric bloating. The prevalence of dry eye symptoms in this specific patient population was found to be 44%. Of the 44% of patients experiencing symptomatic dry eyes, 19% rated the severity as present but not bothersome, 13% rated the dry eyes as somewhat bothersome, and 12% rated it as very bothersome. Although the study fails to consider pre-existing dry eye conditions prior to admissions; the association between dry eyes and the development of DED is a constant issue in terms of patient’s experience throughout their hospitalization and compliance with home therapy.

Matossian et al. [12] found that the prevalence rate (PR) and incidence rate (IR) of DED were significantly higher in CPAP users than in the general population. In a most recent retrospective study, data were obtained from IBM MarketScan® Commercial and Medicare Supplemental claims database (IBM Corp., Armonk, NY). Adults with more than one claim of CPAP and a diagnosis of sleep apnea were found to have an increase in both PR and IR increased over a three-year period. PRs for the first, second, and third years of continuous CPAP use were 6.2%, 10.0%, and 13.0%, respectively. IRs for the first, second, and third years were 4.0%, 7.3%, and 10.3%, respectively. This suggested that an increase in the duration of CPAP therapy was positively correlated with the development of DED. Interestingly, the data also suggested that females and older patients were more likely to develop DED with NIV use than male and younger patients. The potential associations of immunologic or metabolic disorders associated with gender and age could play a significant role in predisposing patients to developing dry eye symptoms while on NIV therapy. These factors can be explored further in future studies to help guide NIV therapy and implement more aggressive ophthalmologic protocols in populations more at risk of developing DED.

One study showed patients who received NIV therapy in the ICU for acute pulmonary exacerbation experienced ocular dryness leading to irritation as a complication, among other more common side effects such as discomfort, pressure ulcers, and mouth dryness. Yesilbalkan and Ozbudak [17] utilized a novel NIV-related complications questionnaire to document such side effects. It recorded the occurrence rate of dry eyes on the first day (65% of patients), fourth day (70% of patients), and seventh day (67.5% of patients) of NIV therapy. Patients experienced eye dryness throughout the duration of treatment, and there was no significant increase in severity with the progression of days. This study suggests that eye dryness is a potential side effect of therapy, but the duration of treatment during the acute setting is unlikely to increase the severity. Thus, preventative measures or proper eye care management will help reduce the initial occurrence of ocular dryness in these patients.

In a two-phase prospective cohort study, Kousha et al. [14] studied 371 adult ICU patients from the Royal Cornwall Hospitals NHS Trust (Cornwall, United Kingdom). Ophthalmic assessments were performed on patients, which included a full eye examination of the external eye, eyelids, eyelid position, and ocular surface using a portable slit lamp. In the first phase, the risk factors for and the rate of exposure keratopathy (EK) were measured in 257 patients. EK is corneal damage due to excessive ocular dryness exhibited in patients suffering from DED [21]. The total rate of EK was found to be 21% in mechanically ventilated patients, which represent both invasive and non-invasive. Based on the data in phase 1, an eye care protocol was developed and implemented during phase 2 with consideration for how often Lacri-Lube® lubricant eye ointment should be used based on eye assessments performed at a minimum every eight hours. After protocol implementation, Kousha et al. found a significant reduction in keratopathy rates. In phase 2, only 2.6% of patients developed EK compared to the 21% rate of EK in phase 1. This suggests that the use of any form of mechanical ventilation increases the incidence of ocular irritation and dryness and that proper eye care could significantly reduce complications.

NIV usage subjects the unprotected eye to mechanically generated air. The physical insult of excessive drying disrupts the eye’s natural tear film, which acts as a barrier against pathogenic infection [22]. Some patients reportedly use tape to prevent airflow from the mask to the eyes, but this not only fails to resolve the dry eye symptoms but may also exacerbate the ocular symptoms by causing secondary lagophthalmos, incomplete closure of the eyelids, due to mechanical ectropion [23]. The discomfort associated with DED may lead to increased eye rubbing, which further promotes fomite transmission, irritation, and worsening of ocular symptoms. In the inpatient setting, health care providers should implement proper eye care protocols to reduce the incidence of ocular dryness [14]. In the outpatient setting, physicians should consider educating patients on ways to prevent excessive drying with prolonged NIV use, especially patients with a history of DED. Patients should wear properly fitted masks that reduce the upward flow of air toward the eyes. Providers should prescribe lubricating eye drops and emollients that can preserve the protective tear film. Blinking exercises may also be beneficial in some cases. Special attention should be paid to individuals who have a history of keratopathy, recent ophthalmic surgery, or other inflammatory or autoimmune diseases that can further worsen dry eyes and its associated symptoms [24]. The use of oral omega-3 fatty acids found in fish oil or flaxseed oil can help dry eyes by increasing the quality of meibum in meibomian gland dysfunction. Additionally, omegas reduce ocular surface inflammation by altering the polar lipid profile and decreasing the saturated fatty acid concentration of meibomian gland secretion [19].

## Conclusions

The etiology of keratoconjunctivitis sicca (DED) is multifactorial, but research has shown that the use of NIV in treating OSA, COPD exacerbation, and respiratory failure has contributed to an increased incidence. Prolonged use of improperly fitted masks accompanying NIV devices accelerates ocular drying, causing irritation and inflammation and increasing the risk of infection and other ocular pathologies. The adverse effects of NIV use in causing dry eye symptoms and the development of DED may cause patient dissatisfaction leading to possible noncompliance with home NIV therapy. The use of NIV is an important part of treatment for many respiratory pathologies; therefore, so patient compliance is paramount. Thus, proper patient education, implementation of behavioral modifications, and proper eye care protocols, as discussed above, can help reduce the undesirable effect of dryness and its subsequent effects. Future studies can be focused on different preventative measures and therapies that can decrease the occurrence of dry eye symptoms during NIV usage, accessing the success rates of each remedy introduced while on NIV therapy.

## References

[1] Pierson DJ (2009). History and epidemiology of noninvasive ventilation in the acute-care setting. Respir Care.

[2] Rotenberg BW, Murariu D, Pang KP (2016). Trends in CPAP adherence over twenty years of data collection: a flattened curve. J Otolaryngol Head Neck Surg.

[3] Bommert CM, Grupcheva CN, Radeva MN, Grupchev DI, Boyadzieva MR (2020). Sleep apnea and dry eye: how sleep apnea affects the eye surface. Ophthatherapy.

[4] Moss SE, Klein R, Klein BE (2000). Prevalence of and risk factors for dry eye syndrome. Arch Ophthalmol.

[5] Messmer EM (2015). The pathophysiology, diagnosis, and treatment of dry eye disease. Dtsch Arztebl Int.

[6] Holly FJ, Lemp MA (1977). Tear physiology and dry eyes. Surv Ophthalmol.

[7] Mertzanis P, Abetz L, Rajagopalan K (2005). The relative burden of dry eye in patients' lives: comparisons to a U.S. normative sample. Invest Ophthalmol Vis Sci.

[8] Alves M, Novaes P, Morraye Mde A, Reinach PS, Rocha EM (2014). Is dry eye an environmental disease?. Arq Bras Oftalmol.

[9] Santos M, Hofmann RJ (2017). Ocular manifestations of obstructive sleep apnea. J Clin Sleep Med.

[10] Stapleton F, Alves M, Bunya VY (2017). TFOS DEWS II Epidemiology Report. Ocul Surf.

[11] Farrand KF, Fridman M, Stillman IÖ, Schaumberg DA (2017). Prevalence of diagnosed dry eye disease in the United States among adults aged 18 years and older. Am J Ophthalmol.

[12] Matossian C, Song X, Chopra I, Sainski-Nguyen A, Ogundele A (2020). The prevalence and incidence of dry eye disease among patients using continuous positive airway pressure or other nasal mask therapy devices to treat sleep apnea. Clin Ophthalmol.

[13] Hayirci E, Yagci A, Palamar M, Basoglu OK, Veral A (2012). The effect of continuous positive airway pressure treatment for obstructive sleep apnea syndrome on the ocular surface. Cornea.

[14] Kousha O, Kousha Z, Paddle J (2018). Exposure keratopathy: Incidence, risk factors and impact of protocolised care on exposure keratopathy in critically ill adults. J Crit Care.

[15] Pépin JL, Leger P, Veale D, Langevin B, Robert D, Lévy P (1995). Side effects of nasal continuous positive airway pressure in sleep apnea syndrome. Study of 193 patients in two French sleep centers. Chest.

[16] Smith TA, Ingham JM, Jenkins CR (2019). Respiratory failure, noninvasive ventilation, and symptom burden: an observational study. J Pain Symptom Manage.

[17] Yesilbalkan Yesilbalkan, OU OU, Ozbudak G (2019). Noninvasive mechanical ventilation related some complications: patients treating intensive care unit. Int J Caring Sci.

[18] Sullivan CE, Issa FG, Berthon-Jones M, Eves L (1981). Reversal of obstructive sleep apnoea by continuous positive airway pressure applied through the nares. Lancet.

[19] Chhadva P, Goldhardt R, Galor A (2017). Meibomian gland disease: the role of gland dysfunction in dry eye disease. Ophthalmology.

[20] Chang VT, Hwang SS, Kasimis B, Thaler HT (2004). Shorter symptom assessment instruments: the Condensed Memorial Symptom Assessment Scale (CMSAS). Cancer Invest.

[21] Kuruvilla S, Peter J, David S (2015). Incidence and risk factor evaluation of exposure keratopathy in critically ill patients: a cohort study. J Crit Care.

[22] Sun CB, Wang YY, Liu GH, Liu Z (2020). Role of the eye in transmitting human coronavirus: what we know and what we do not know. Front Public Health.

[23] Vallabhanath P, Carter SR (2000). Ectropion and entropion. Curr Opin Ophthalmol.

[24] Moshirfar M, West WB Jr, Marx DP (2020). Face mask-associated ocular irritation and dryness. Ophthalmol Ther.

